# Effects of serum sodium and chloride levels in the outcome of critically ill pediatric patients in the post-operative period of liver transplantation

**DOI:** 10.1186/s12882-023-03195-1

**Published:** 2023-05-22

**Authors:** Michele Luglio, Werther B. de Carvalho, Uenis Tannuri, Ana Cristina A. Tannuri, Rodrigo Hideki Matsura, Gardenia Morais França, Artur F. Delgado

**Affiliations:** 1 Pediatric Critical Care Center, Instituto da Criança e do Adolescente do Hospital das Clínicas da Faculdade de Medicina da Universidade de São Paulo, São Paulo, SP Brazil; 2 Pediatric Surgery and Liver Transplant Team, Instituto da Criança e do Adolescente do Hospital das Clínicas da Faculdade de Medicina da Universidade de São Paulo, São Paulo, SP Brazil

**Keywords:** Sodium, Chloride, Liver transplant, Acute renal failure

## Abstract

**Background:**

Sodium and chloride disturbances have attracted increasing attention in recent years. Many pathophysiological effects are associated with hyperchloremia, including reduction in mean arterial pressure and acute renal disease. Pediatric patients undergoing liver transplantation are at risk of developing various electrolyte and biochemical abnormalities, with an impact on their postoperative outcomes.

**Objective:**

To analyze the impacts of serum sodium and chloride levels on prognosis of Pediatric Liver Transplant receptors.

**Methods:**

This was a retrospective analytical observational study performed in a single transplant reference center in Sao Paulo, Brazil. Included patients were pediatric patients who underwent liver transplantation between January 2015 and July 2019. Statistical regression analysis and General Estimating Equations analysis were performed to evaluate the impacts of sodium and chloride disturbances on the development of acute renal failure and mortality.

**Results:**

A total of 143 patients were included in this study. The main diagnosis was Biliary Atresia (62.9%). Twenty-seven patients died (18.9%), and graft dysfunction was the main cause of death (29.6%). The only variable individually associated with 28-days mortality was PIM-3 score (HR 1.59, CI 95% 1.165–2.177, p = 0.004). Forty-one patients (28.6%) developed moderate or severe AKI. PIM-3 score (OR 3.052, 95% CI 1.56–5.97, p = 0.001), hypernatremia (OR 3.49, 95% CI 1.32–9.23, p = 0.012), and hyponatremia (OR 4.24, 95% CI 1.52–11.85, p = 0.006) were independently associated with the development of moderate/severe AKI.

**Conclusions:**

In pediatric patients after liver transplantation, PIM-3 score, and abnormal serum sodium levels were correlated with AKI development.

## Introduction

In recent years, the prognostic effects of serum chloride disturbances have gained increasing attention in intensive care. Chloride is the main anion in the human body and is responsible for 2/3 of the plasmatic negative charges [1]. Its role in acid-base equilibrium, muscular activity, and immunomodulation is not yet fully understood, despite chloride abnormalities being present in nearly 25% of critically ill adult patients [2].

Among the different pathophysiological effects associated with hyperchloremia, pro-inflammatory effects [3, 4], reduction of mean arterial pressures [5], acute renal disease [6] and erythropoiesis inhibition [4, 7], have been further studied in critically ill patients.

In children, data regarding the effects of chloride and other electrolyte abnormalities have been recently studied in specific and critically ill populations [8–11]. Stenson et al. [8] evaluated the effects of serum chloride levels ([Cl]) in patients with septic shock admitted to the pediatric intensive care unit (PICU). In this study, a [Cl]_minimum_ > 110 mEq/L was positively associated with higher mortality ratios (OR 3.7; CI95% = 2.0-6.8; p < 0.001).

Dysnatremias are common events in the intensive care population. Hyponatremia is the most common electrolyte disorder in hospitalized patients, with a prevalence of 22% [12]. Although described as less frequent, hypernatremia is associated with higher hospital mortality, with studies pointing to mortality ratios between 30 and 48% in patients with serum sodium concentrations above 150 mEq/L [13]. In liver transplant patients, the growing interest in serum sodium disturbances arise from observations that pre-transplant dysnatremias leads to worst prognosis after transplantation [14]. Knowledge towards the association of post-transplant serum sodium imbalance with prognosis, especially on pediatric patients, is still scarce.

Pediatric patients undergoing liver transplantation, given the surgical complexity, preoperative characteristics, and postoperative complications, are at risk for organic dysfunctions and a complicated clinical course [15, 16]. These patients typically present large volemic shifts in the immediate postoperative period due to the administration of large volumes of crystalloids and blood products during and after surgery [15]. Therefore, there is great interest in understanding the biochemical and electrolyte pattern of this group of patients as well as its impact on postoperative prognosis.

The aim of this study was to evaluate the potential association of sodium and chloride levels through the postoperative period of pediatric liver transplant recipients, and its impact on prognosis, notably the development of acute kidney injury (AKI) and mortality.

## Materials and methods

### Study design and participants

We performed a retrospective analytical observational study of pediatric patients who underwent liver transplantation between January 2015 and July 2019 at the Instituto da Criança e do Adolescente do Hospital das Clínicas da Faculdade de Medicina da Universidade de São Paulo (ICR-HCFMUSP), a reference center in Brazil for pediatric liver transplantation.

The inclusion criteria were: (1) age between 1 month and 18 years and (2) admission to the PICU on the immediate postoperative period after liver transplantation. All included patients were previously clinically stable and out of critical illness, being referred to the operation room from home or general infirmaries. Exclusion criteria were as follows: (1) patients with acute hepatic failure and (2) patients who underwent a second liver transplantation. These patients were excluded because of the lack of information regarding previous clinical conditions treated outside the study’s institution and the large number of complication variables related to these two conditions.

The outcomes of interest analyzed were: (1) adjusted hazard-ratios for mortality over the first 28 days of PICU and (2) development of new Kidney Disease Improving Global Outcomes (KDIGO) [17] stages 2 and 3 acute kidney injury (AKI). Both hourly urine output and worst daily serum creatinine levels were used for AKI diagnosis and stratification.

Hospital das Clínicas da Faculdade de Medicina da Universidade de Sao Paulo Ethics’ Committee – CEP (Approval number #1484/04/2019) previously approved the present study. All methods were carried out in accordance with ethical guidelines and regulations, and in accordance with the Helsinki Declaration. Due to the retrospective nature of the study, the application of written informed consent was waived by Hospital das Clínicas da Faculdade de Medicina da Universidade de Sao Paulo Ethics’ Committee - CEP.

### Variables and definitions

Patient demographic and clinical data were obtained from electronic medical records and service databases. Clinical and laboratory data were collected through the PICU and evaluated at previously defined time points: admission, postoperative day 1 (D1), postoperative day 3 (D3), postoperative day 5 (D5), post-operative day 14 (D14), and discharge or death.

Baseline variables of interest were as follows: (1) age; (2) sex; (3) weight; (4) baseline diagnosis of hepatopathy; (5) type of transplantation (living or deceased donor); (6) absolute and relative size of the organ implant (defined as implant-to-body weight in %); (7) baseline creatinine values; (8) Pediatric End-Stage Disease Score (PELD) [18]; (8) intra-operatory fluid balance; and (9) Pediatric Index of Mortality 3 score (PIM-3) [19].

Baseline serum creatinine values were obtained from hospital admission laboratorial exams obtained immediately before liver transplantation. Routinely, all the patients admitted for liver transplantation in the study’s institution are submitted to a baseline laboratorial screening immediately before operation room admission for liver transplantation.

Sequentially, at the above-mentioned predefined time points, serial measures of biochemical and organic functional markers were collected: (1) serum chloride, (2) arterial pH and bicarbonate levels, (3) serum sodium, (4) serum potassium, (5) albumin levels, (6) albumin-corrected anion-gap (AG), (7) aspartate aminotransferase (AST), (8) serum creatinine (Cr), and (9) KDIGO acute kidney injury category.

For hyperchloremia, the study used [Cl] $\ge$ 110 mEq/L. Hyponatremia was defined as [Na] < 135 mEq/L and hypernatremia was defined as [Na] > 145 mEq/L.

For data collection and management, preconceived RedCap® (Research Electronic Data Capture) data tools hosted in our institution were used.

### Statistical analysis

Quantitative variables are described as means and standard deviations or medians and interquartile ratios. Qualitative and demographic variables were reported as absolute and relative frequencies. Continuous variables with normal distribution were initially evaluated using the Student’s t-test. Continuous variables without a normal distribution were analyzed using the Mann–Whitney test. Categorical variables were analyzed using chi-squared, Fischer’s exact test, or likelihood ratio tests, when applicable.

For Hazard-ratios for mortality in 28 days, the influences of baseline parameters were evaluated individually by bivariate Cox regression, including age, sex, weight, presence of hyperchloremia on PICU admission, presence of hyper or hyponatremia during PICU stay, estimated intraoperative fluid balance, baseline serum creatinine levels, PIM-3 predicted mortality, PELD/MELD scores, and type of liver donor (living or deceased). The multiple Cox regression models were adjusted for each outcome, the variables that presented a descriptive level lower than 0.20 (p < 0.20) in the bivariate analysis, other than sex, age, and presence of hyperchloremia on admission, were maintained in the complete adjusted model. Final complete model included age, sex, presence of hyperchloremia on PICU admission, PIM-3 predicted mortality and presence of hypo or hypernatremia during PICU stay.

For the evolution with KDIGO 2 or 3 (moderate/severe) AKI, associations of the qualitative variables with the outcomes were analyzed using the chi-squared or Fischer’s exact test, and for continuous variables by the Mann-Whitney test, with the influence of isolated parameters on the outcome being evaluated by bivariate logistic regressions including age, sex, weight, presence of hyperchloremia on PICU admission, presence of hyper or hyponatremia during PICU stay, estimated intraoperative fluid balance, baseline serum creatinine levels, PIM-3 predicted mortality, PELD/MELD scores, and type of liver donor (living or deceased). The models of multiple logistic regression were adjusted with the variables that presented a descriptive level on bivariate analysis of less than 0.20 (p < 0.20), other than sex, age, and presence of hyperchloremia on PICU admission. Final complete model included age, sex, presence of hyperchloremia on PICU admission, PIM-3 predicted mortality, PELD/MELD scores, type of liver donor and presence of hypo or hypernatremia during PICU stay.

Linear correlations between serum sodium (maximum and minimum) and admission chloride were analyzed through Pearson’s correlation and represented by Pearson’s correlation coefficient (r), as a measure of correlation strength.

Longitudinal data collected throughout the PICU length of stay were described by time point of evaluation and by main outcome status (survivors vs. non-survivors) and compared with generalized estimating equations (GEE) with marginal-normal distribution and identity link functions, assuming an auto-regressive correlation matrix of first order between the time points.

A type 1 error of 5% was considered for statistical significance.

Statistical analyses were performed on specific software, including SPSS (IBM SPSS Statistics®) and SAS (SAS Institute®).

## Results

**Fig. 1:**
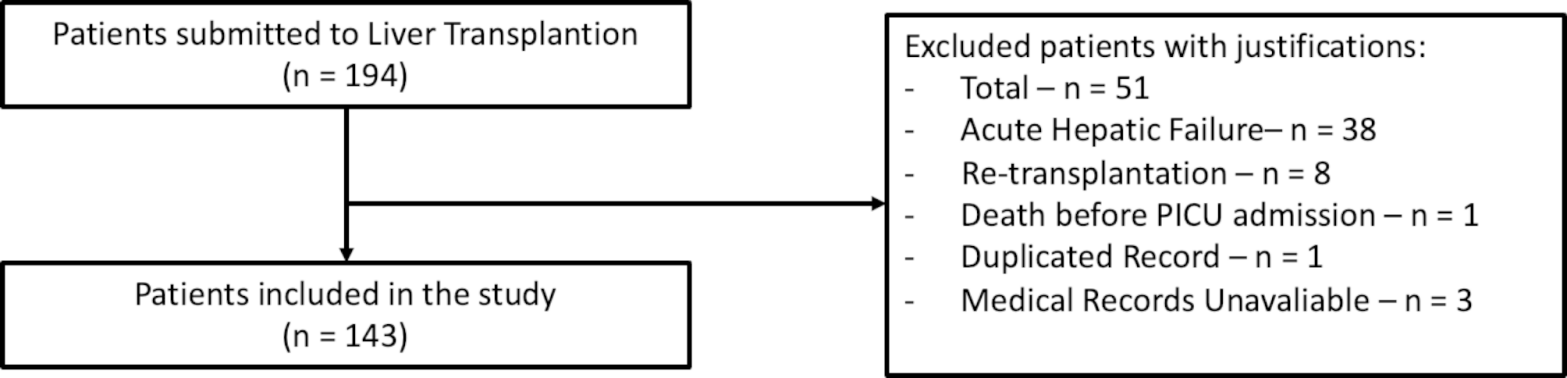
Detailed inclusion and exclusion process:

A total of 194 patients underwent liver transplantation at the study center between January 2015 and July 2019. Based on the inclusion and exclusion criteria, 143 patients were included in the study. Figure 1 shows the details of the inclusion/exclusion process.

The general characteristics of the study groups are presented in Table 1. Patients were divided into groups based on the presence or absence of hyperchloremia on PICU admission after liver transplantation.

**Table 1:** General Characteristics of Study Population according to the presence/absence of hyperchloremia on PICU admission:

Variable	Group		Total (N = 143)	p-value
	Non-hyperchloremia (N = 62)	Hyperchloremia (N = 81)		
**Age (months)**				
Median (min; max)	13.5 (4.7; 192.8)	14.2 (5.7; 185.8)	14 (4.7; 192.8)	0.310
**Sex**				
Female, n (%)	29 (46.8)	47 (58)	76 (53.1)	0.182
**Weight (Kg)**				
Median (min; max)	10 (5.5; 73)	8.4 (4.3; 50)	9 (4.3; 73)	0.053
**Type of Donor**				
Living, n (%)	49 (79)	70 (86.4)	119 (83.2)	0.241
**Primary Diagnosis, n (%)**				
Biliary Atresia, n (%)	32 (51.6)	58 (71.6)	90 (62.9)	0.010*
Malignancies, n (%)	2 (3.2)	2 (2.5)	4 (2.8)	
Metabolic Diseases, n (%)	5 (8.1)	6 (7.4)	11 (7.7)	
Cholestatic Diseases, n (%)	9 (14.5)	3 (3.7)	12 (8.4)	
Auto-imune hepatites, n (%)	5 (8.1)	6 (7.4)	11 (7.7)	
Other, n (%)	9 (14.5)	12 (14.8)	21 (14.7)	
**Graft-to-Body Ratio (%)**				
Mean (SD)	3.1 (1.27)	3.44 (1.28)	3.29 (1.28)	0.128
**Intraoperatory Fluid Balance (mL/kg)**				
Median (min; max)	155 (-100; +627)	136 (+ 9; +585)	147.5 (-100; +627)	0.771
**PELD/MELD score**				
Median (min; max)	16.4 (0; 39)	15.7 (0; 40.1)	15.9 (0; 40.1)	0.442
**PIM-3 predicted mortality (%)**				
Median (min; max)	1.28 (0.74; 3.46)	1.34 (0.78; 5.82)	1.33 (0.74; 5.82)	0.494
**Baseline Serum Creatinine (mg/dL)**				
Median (min; max)	0.3 (0.17; 3.57)	0.17 (0.16; 0.57)	0.21 (0.16; 3.57)	< 0.001**
**Length of Stay in PICU (days)**				
Median (min; max)	11 (3; 92)	13 (0;45)	11 (0; 92)	0.886
**Days of Mechanical Ventilation (days)**				
Median (min; max)	1 (0; 22)	1 (0; 28)	1 (0; 28)	0.407
**Need of Renal Replacement Therapy, n (%)**				
Yes, n (%)	7 (11.3%)	11 (13.6%)	18 (12.6%)	0.682
**Maximum sodium (mEq/L)**				0.822
Mean (SD)	141.9 (3.5)	142 (3.7)	142 (3.6)	
Median (min; max)	142 (134; 149)	141 (134; 154)	141 (134; 154)	
**Minimum sodim (mEq/L)**				0.528
Mean (SD)	132.7 (5.9)	132.1 (5.6)	132.3 (5.7)	
Median (min; max)	133 (119; 149)	134 (111; 140)	134 (111; 149)	

Only two patients included in the analysis showed levels of admission serum chloride lower than 96 mEq/L. The admission serum chloride levels varied between 92 and 126 mEq/L on included patients. The study groups differed in their primary diagnosis distribution, with a higher frequency of biliary atresia among patients with PICU admission hyperchloremia (71.6% vs. 51.6%). The primary diagnosis was more varied among patients without hyperchloremia on admission. Baseline serum creatinine levels were lower in patients with hyperchloremia on PICU admission (0.17 (0.16, 0.57); p < 0.001).

A total of 27 patients died: 15 in the hyperchloremia group (18.5%) and 12 in the non-hyperchloremia group (19.4%). The main causes of death are shown in Table 2. The main cause of death was primary graft dysfunction (n = 8; 29.6%).

**Table 2:** Distribution of Causes of Death According to presence or absence of Hyperchloremia on PICU Admission:

Variable	Group		Total (N = 27)	p-value	
	Non-hyperchloremia (N = 12)	Hyperchloremia (N = 15)			
Septic Shock, n (%)	2 (16.7%)	4 (26.7%)	6 (22.2%)	0.066*	
Graft Primary dysfunction, n (%)	4 (33.3%)	4 (26.7%)	8 (29.6%)		
Respiratory Failure / PARDS, n (%)	1 (8.3%)	6 (40.0%)	7 (25.9%)		
Intracranial Hypertension, n (%)	3 (25.0%)	0 (0%)	3 (11.1%)		
Acute Renal Failure / Electrolyte Disorders, n (%)	2 (16.7%)	1 (6.7%)	3 (11.1%)		

Table 3 shows the unadjusted and adjusted Hazard-ratios (HR) for 28-days mortality. Only the value of PIM-3 predicted mortality showed a statistically significant influence on mortality, maintained after adjustment for confounding variables, with the risk of mortality demonstrating an elevation of 59.2% for each 1% of PIM-3 predicted mortality.

**Table 3:** Adjusted and Non-adjusted Hazard Ratios (HR) for 28-day mortality (bivariate and multiple Cox Regression):

Variable	Unadjusted HR	CI 95%			p	Adjusted HR		CI 95%		p	
		Inferior		Superior				Inferior	Superior		
Age (months)	0.998	0.989		1.006	0.588	0.998	0.988		1.007	0.634	
Sex (Male)	1.772	0.809		3.883	0.153	1.852	0.810		4.235	0.144	
Weight (kg)	0.992	0.952		1.032	0.682						
Hyperchloremia on PICU Admission	1.128	0.517		2.461	0.761	1.003	0.428		2.349	0.995	
PIM-3 Predicted Mortality (%)	1.749	1.315		2.327	**< 0.001**	1.592	1.165		2.177	**0.004**	
PELD/MELD	1.011	0.971		1.053	0.593						
Type of Donor (Living)	0.698	0.279		1.745	0.442						
Intraoperatory Fluid Balance (mL/kg)	1.000	0.997		1.003	0.787						
Baseline Serum Creatinine (mg/dL)	0.910	0.305		2.718	0.866						
Hypernatremia on PICU	1.878	0.848		4.158	0.120	1.418	0.570		3.527	0.453	
Hyponatremia on PICU	0.554	0.244		1.258	0.158	0.810	0.315		2.087	0.663	

Forty-one patients (28.6%) developed moderate/severe AKI after the PICU stay. In the non-adjusted analysis, the presence of hypernatremia throughout PICU stay, the presence of hyponatremia throughout PICU stay and PIM-3 predicted mortality showed a statistically significant correlation with the evolution of moderate/severe acute kidney injury (AKI) as defined by KDIGO categories 2 and 3. These effects were maintained after adjusting for baseline characteristics and confounding variables on logistic multiple regression analysis, as shown in Table 4. Each 1% increase in PIM-3 predicted mortality led to a 2.05-times increase in the risk of moderate/severe AKI, independently.

**Table 4:** Odds-ratio (OR) for the development of moderate/severe AKI, adjusted and non-adjusted for baseline characteristics (multiple logistic regression model):

Variable	Unadjusted OR	CI 95%		p	Adjusted OR	CI 95%			p	
		Inferior	Superior			Inferior		Superior		
Age (months)	1.004	0.996	1.011	0.577	1.001	0.991		1.011	0.860	
Sex (Male)	1.28	0.62	2.64	0.507	1.50	0.64		3.50	0.350	
Weight (kg)	1.012	0.980	1.044	0.401						
Hyperchloremia on PICU Admission	0.95	0.89	1.02	0.229	0.68	0.29		1.61	0.377	
PIM-3 Predicted Mortality (%)	2.527	1.410	4.529	**0.003**	3.052	1.560		5.970	**0.001**	
PELD/MELD	1.032	0.993	1.073	0.072	1.027	0.980		1.076	0.260	
Type of Donor (Living)	0.49	0.20	1.22	**0.022**	0.50	0.16		1.61	0.246	
Intraoperatory Fluid Balance (mL/kg)	0.998	0.995	1.001	0.321						
Baseline Serum Creatinine (mg/dL)	2.439	0.653	9.102	0.525						
Hypernatremia on PICU	2.59	1.13	5.94	**0.022**	3.49	1.32		9.23	**0.012**	
Hyponatremia on PICU	2.87	1.27	6.45	**0.009**	4.24	1.52		11.85	**0.006**	

Pearson’s correlation coefficient was performed comparing admission serum chloride levels, maximum and minimum sodium levels through the observation period. No statistically significant correlation was found between admission chloride and maximum sodium levels (r = 0.083 – p = 0.325) nor between admission chloride and minimum sodium levels (r = -0.020 – p = 0.809).

**Fig. 2:**
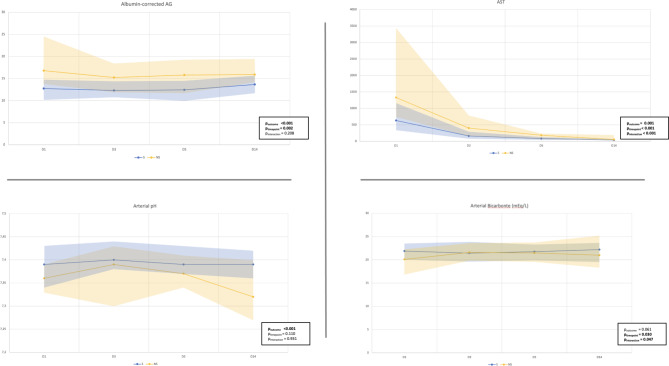
Evolution curves of median arterial pH, arterial bicarbonate, AST, and Albumin-corrected AG on the different timepoints by groups (S = Survivors; NS = non-survivors). Shaded areas show interquartile ratio:

Biochemical variables were evaluated longitudinally with respect to their relationship with mortality on 28-days and at each postoperative time point. Table 5 demonstrates that the mean tendency of arterial bicarbonate measures and serum AST among patients who died was statistically different over time from that of the survivors (p_interaction_ = 0.047; p_intercation_ = 0.001, respectively). Arterial pH values were significantly higher in survivors at all analyzed time points (p < 0.001). Non-survivors showed a higher albumin-corrected AG independent of the analyzed time point. These differences are graphically shown in Fig. 2. Collinearity between admission arterial bicarbonate values and admission chloride values was tested by Pearson’s correlation. No significant correlation was found (r = 0.002 – p = 0.9755).

**Table 5:** Longitudinal Biochemical variables evaluated during PICU length-of-stay, on different timepoints (GEE with marginal-normal distribution and identity link functions with AR correlation matrix of first order):

Variable	Post-operative Days								p (outcome)	p (timepoint)	p (interaction)
	D1		D3		D5		D14				
**Sodium (mEq/L)**									0.990	**< 0.001**	0.237
Survivors	140 (127; 154)		137 (119; 146)		136 (119; 145)		137 (125; 154)				
median (min; max)											
Non-survivors	141 (127; 154)		137 (122; 140)		137.5 (124; 147)		137 (120; 149)				
median (min; max)											
**Potassium (mEq/L)**									0.272	0.728	0.147
Survivors	4 (2.5; 6.4)		4.1 (2.8; 5.2)		4.25 (3.1; 5.8)		4 (2.6; 5.8)				
median (min; max)											
Non-survivors	4.4 (3.1; 7.4)		4 (2.6; 5.2)		3.8 (2.5; 10.1)		4.3 (2.8; 6.6)				
median (min; max)											
**Arterial pH**									**< 0.001**	0.110	0.931
Survivors	7.39 (7.07; 7.52)		7.40 (7.08; 7.54)		7.39 (7.00; 7.53)		7.39 (7.23; 7.49)				
median (min; max)											
Non-survivors	7.36 (6.65; 7.45)		7.39 (7.18; 7.52)		7.37 (6.59; 7.56)		7.32 (7.14; 7.48)				
median (min; max)											
**Arterial Bicarbonate (mEq/L)**									0.061	**0.030**	**0.047**
Survivors	22 (11.4; 27.4)		21.5 (12.5; 29.7)		21.8 (13.8; 32.9)		22.2 (13.5; 28.1)				
median (min; max)											
Non-survivors	20.1 (6.1; 28)		21.6 (15.7; 30.9)		21.5 (3.4; 31.2)		21 (15.6; 27.1)				
median (min; max)											
**Albumin-corrected AG**									**< 0.001**	**0.002**	0.208
Survivors	12.8 (4.5; 29.6)		12.3 (3.2; 21)		12.5 (2.3; 22)		13.7 (0.9; 22.4)				
median (min; max)											
Non-survivors	16.8 (5.4; 51.8)		15.3 (8.3; 34.6)		15.8 (5.6; 51.6)		15.9 (9.4; 29.6)				
median (min; max)											
**Plasmatic Albumin (g/dL)**									0.747	0.248	0.113
Survivors	3.1 (1.3; 4.4)		2.95 (2.1; 4.0)		2.95 (2.2; 4.5)		2.9 (1.7; 4.0)				
median (min; max)											
Non-survivors	2.9 (1.8; 4.0)		3.0 (2.1; 4.5)		2.95 (1.6; 4.7)		3.0 (2.1; 4.0)				
median (min; max)											
**AST (U/dL)**									**0.001**	**< 0.001**	**< 0.001**
Survivors	633.5 (28; 12,087)		162.5 (18; 2,694)		82.5 (1.7; 934)		40 (9; 953)				
median (min; max)											
Non-survivors	1,328 (196; 9,223)		403 (44; 2,169)		183.5 (22; 600)		54 (16; 1,147)				
median (min; max)											

## Discussion

Our group found that hyperchloremia on PICU admission after liver transplantation was not associated with mortality or the development of moderate/severe AKI in pediatric patients. In the same group of patients, the presence of hypernatremia and hyponatremia throughout the PICU stay and the PIM-3 predicted mortality risk were associated with the development of AKI (KDIGO 2 and 3).

The clinical effects of hyperchloremia are of new interest in the intensive care community [20], led by the discussion of the potentially beneficial use of balanced-crystalloid solutions for fluid resuscitation [21]. Studies in adult septic patients have suggested positive impacts on various outcomes with the use of this type of solution in the resuscitation phases of septic shock [22, 23]. However, there is still some controversy, especially when evaluating different groups of patients, with different critical diagnoses, and among different age groups.

Oh et al. [10] retrospectively analyzed the medical records of 7,991 critically ill surgical patients, demonstrating that exposure to hyperchloremia during the first 3 days of ICU stay or the ascension of serum chloride levels of more than 6 mEq/L did not correlate with a higher incidence of AKI (OR 1.09 CI95% 0.80–1.49, p = 0.571). The only study analyzing hyperchloremia in post-liver transplant patients was performed by Nadeem et al. [25], with 158 post-transplant patients, which showed that higher volumes of chloride-rich expansion fluids and hyperchloremia on post-operative day 2 were associated with the development of AKI.

The results of observational studies [8–11] are, nonetheless, conflicting. The main issues are related to different methodologies for the evaluation of hyperchloremia and other electrolytes, such as varying cut-off values and time points for analysis. In our study, we addressed this situation by previously defining the desired cut-off of serum chloride and serum sodium, by establishing a fixed time point for evaluation of the clinical effects, and by performing a longitudinal analysis based on GEE, a statistical tool used for sequential measurements.

In our study, admission hyperchloremia was not associated with AKI or mortality in critically ill post-liver transplantation patients, even after adjustment for potential confounding variables. Our observations may be related to some particularities of our population, such as the standardized use of balanced solutions as intraoperative fluid, leading to lower overall levels of serum chloride, as well as a lack of statistical power to show differences due to the small study population. Additionally, the total chloride load administered to the patients was not assessed because of a lack of data, particularly during surgery.

The incidence of moderate/severe AKI in our study (28.6%) was similar to that described in previous pediatric studies [21]. When analyzing other parameters, the presence of abnormal serum sodium levels and PIM-3 predicted mortality were correlated with a higher incidence of AKI. A previous study by Ferah et al. [26] showed that the development of AKI was correlated with higher serum sodium and lower plasma albumin levels. In the post-liver transplant setting, the development of AKI represents a surrogate marker for the worst clinical course [24, 26], what makes it important to detect potential early biomarkers for this evolution. Due to the retrospective nature of the study, however, it is possible only to establish correlations but not causality. In this context, future prospective studies are needed to evaluate potential biomarkers for AKI and the complicated clinical course of pediatric liver transplant patients.

Dysnatremias are electrolyte disorders associated with AKI. Low levels of serum sodium can arise as an effect of reduced capacity of the kidney to excrete electrolyte-free water in the context of renal injury. Although less frequent than hyponatremia, high values of serum sodium concentrations can appear on the non-oliguric phase of AKI or during the recovery of acute tubular necrosis, both situations where free water excretion and urine concentration capacities may be compromised [27, 28]. The association of sodium imbalances and AKI is of particular importance in post-liver transplant patients, on which serum creatinine levels may not be ideal to an early detection of renal injury due to many interferents. Further studies are needed to better stablish causality and precise time correlation between dysnatremias and AKI in the context of pediatric liver transplantation.

While PIM-3 does not contemplate a direct assessment of kidney function on the score composition, our study shows that it performed well on determining a higher risk of moderate and severe AKI. Even when compared to other biomarkers, such as admission hyperchloremia and direct scores for determining liver dysfunction (MELD/PELD), PIM-3 had a higher capacity of determining a worse renal prognosis on PICU stay. These observations may indicate that the score is suitable for renal failure risk stratification on post-liver transplant pediatric patients, helping to raise awareness to this frequent type of organ failure in such group of critical patients.

Analysis of longitudinal biochemical markers showed that arterial pH, albumin-adjusted AG, and AST levels differed between survivors and non-survivors. Non-survivors showed a tendency towards higher AST levels at all time points, associated with peak initial values and longer recovery times. These data are consistent with observations from previous studies [29, 30] that analyzed the role of hepatic enzyme assays on the worst prognosis after liver transplantation in adult patients, a phenomenon linked to early graft dysfunction.

Issues related to chloride load and its relation to AKI in post liver transplant recipients, were addressed in a previous study by Nadeem and collaborators [25]. This study showed association between severe AKI and high administration of chloride (more than 3,200 mL of 0.9% saline). The retrospective design and lack of reliable intraoperative data on precise volumes administered to the patients made it impossible to evaluate chloride load in our study. Nonetheless, since the beginning of 2015 the study’s institution intraoperative fluid management protocol for liver transplantation dictated that the preferential crystalloids used for fluid resuscitation would be balanced solutions, such as Ringer-lactate and Plasma-lyte. Despite not being specifically analyzed, it is possible to state that the majority of the crystalloid fluids used during liver transplantation were balanced solutions.

This study has some limitations. First, it was a retrospective study, and, as previously mentioned, the establishment of causality is not possible. Second, the study population was small, which may have compromised the power of the study. Third, the single-center nature of the study may compromise external validity. Fourth, other causes of AKI such as nephrotoxicity were not addressed. Fifth, the serum creatinine levels used may suffer impacts of a substantially positive fluid balance, especially on the first days after liver transplantation. However, strategies to adjust the creatinine levels for the fluid balance are prone to some questions such as the non-exactness of fluid balance estimation in a retrospective study and issues related to the available formulas, which can be subject of biases when considering the correspondence between fluid boluses and serum creatinine concentrations over time [31]. Finally, even with a timeframe of nearly five years, the management of pediatric post liver transplant patients in our institution has not been modified in ways that could impact the study observations.

## Conclusion

In pediatric patients after liver transplantation, hyperchloremia on PICU admission was not related to increased mortality or evolution of AKI. We found that abnormal sodium levels and PIM-3 mortality scores were associated with moderate/severe AKI during the PICU stay. Longitudinal analysis showed that AST and gasometrical variables had different patterns between survivors and non-survivors, with lower levels of AST in the former and higher albumin-adjusted AG in the latter.

## Data Availability

The data that support the findings of this study are available from the Instituto da Criança e do Adolescente do Hospital da Clinicas da Faculdade de Medicina da Universidade de São Paulo, but restrictions apply to the availability of these data, which were used under license for the current study, and so are not publicly available. Data are however available from the authors upon reasonable request and with permission of Instituto da Criança e do Adolescente do Hospital da Clinicas da Faculdade de Medicina da Universidade de São Paulo.

## List of Abbreviations

Acute Kidney Injury: AKI
AG: Anion-gap
AST: Aspartate Aminotransferase
GEE: General Estimating Equations
HR: Hazard-ratio
KDIGO: Kidney Disease Improving Global Outcomes
MELD: Model for End-stage Liver Disease
OR: Odds-ratio
PELD: Pediatric End-Stage Disease Score
PICU: Pediatric Intensive Care Unit
PIM-3: Pediatric Index of Mortality 3 score
[Cl]: Serum chloride concentrations
[Na]: Serum Sodium Concentrations
